# Practices, attitudes and knowledge of midwives and nurses regarding gestational diabetes and pregnancy-induced hypertension

**DOI:** 10.25122/jml-2023-0021

**Published:** 2023-02

**Authors:** Daniela Stan, Claudia Elena Dobre, Doina Carmen Mazilu, Elvira Brătilă

**Affiliations:** 1 Department of Obstetrics and Gynecology, Carol Davila University of Medicine and Pharmacy, Bucharest, Romania; 2 The Order of Nurses, Midwives and Medical Assistants in Romania, Bucharest, Romania; 3 Department of General and Specific Nursing, Carol Davila University of Medicine and Pharmacy, Bucharest, Romania

**Keywords:** midwives, nurses, knowledge, attitudes, practices, gestational diabetes, pregnancy-induced hypertension, GD – Gestational diabetes, Hbp – High blood pressure, M – midwives, M1 – Moment 1 of the assessment, M2 – Moment 2 of the assessment, M3 – Moment 3 of the assessment, ON – Obstetric nurses, PIH – pregnancy-induced hypertension

## Abstract

Midwives (M) and obstetric nurses (ON) play a critical role in providing healthcare for pregnant patients at all stages of pregnancy, and ongoing training and education are essential to ensure the best outcomes. This longitudinal quantitative research study aimed to assess the impact of an educational program on the knowledge, attitudes, and practices of 125 midwives and obstetric nurses regarding care for patients with gestational diabetes and pregnancy-induced hypertension. The original questionnaire consisted of 56 items grouped into 3 subscales assessing knowledge (15 items), attitudes (18 items), and practices (23 items). The questionnaire was administered at three distinct intervals during the educational program: pre-test, post-test, and follow-up at three months. The data were analyzed using ANOVA and Pearson correlation coefficients to determine the significance of the differences between the 3 moments of the administration of the questionnaire. There was a significant increase in the level of knowledge, attitudes, and practices of midwives and obstetric nurses following the training module, which was sustained at 3 months after completion compared to pre-training. The comparative analysis of the total scores for every 3 sets of items revealed the positive impact of the educational program on the level of knowledge, attitudes, and practices of midwives and obstetric nurses.

## INTRODUCTION

Midwives (M) and obstetric nurses (ON) play a critical role in providing primary health care to pregnant patients, and their role is recognized both internationally and nationally. In order to improve the health outcomes of both mothers and children, it is essential to make sure that the quality of their assessment, care, and treatment is at the highest standard. These healthcare providers are deeply engaged in the local community, which allows them to deliver effective interventions that meet the needs of patients, families, and the community at large [1].

The World Health Organization (WHO) emphasized the main priorities for establishing global strategic directions for M and ON in a material focused on 4 areas of interest. These areas include education, jobs, leadership, and service delivery. Adopting and supporting these public policies can lead to increasing the number of M and ON, securing jobs, managing migration, recruiting and retaining M and ON in the areas where they are most needed, developing and strengthening nursing medical leadership in the health and educational systems, and ensuring the respect, protection, and motivation of these categories of medical personnel to obtain their optimal contribution to the provision of health care [2].

Currently, there is a shortage of midwives in hospitals, ambulatory profile units, and community care units in Romania. Unfortunately, a downward trend is estimated in the future as a result of the reduced number of education units that train these specialists.

The COVID-19 pandemic has highlighted the need for midwives in health systems, especially primary health care. The pandemic has underscored the importance of protective measures and investment in all health system specialists involved in healthcare activities, public health services, and the provision of essential medical services [2]. Subsequently, it is essential to implement policies that support, develop, and strengthen the role of midwives in the Romanian health system at the national level. Gestational diabetes (GD) and pregnancy-induced hypertension (PIH) are two common pathologies among pregnant women, especially at extreme ages [3-5]. GD is a transitory metabolic disorder characterized by impaired glucose tolerance during pregnancy, leading to a high glycemic index and serious maternal and fetal complications [6]. The main maternal-fetal complications induced by GD are represented by hydramnios, spontaneous abortion, hypertension, preeclampsia, eclampsia, fetal macrosomia, shoulder dystocia, respiratory distress syndrome, neonatal hypoglycemia and even perinatal mortality [7-8].

Preeclampsia is a hypertensive disorder occurring in the second half of pregnancy characterized by high blood pressure and proteinuria (protein in the urine). This condition affects the main organs in the body, such as the brain, liver, kidneys, or placenta, and can have negative effects on the normal evolution of pregnancy [9].

Screening and preventive care are crucial in minimizing the impact of these pathologies on both the mother and fetus. It is essential that M and ON involved in the care of pregnant women, have the necessary theoretical and practical knowledge to effectively evaluate, diagnose, and manage preventable pathologies during pregnancy. This expertise enables them to intervene effectively in the evaluation, diagnosis, and management of the pregnant patient, providing effective healthcare and reducing infant and maternal mortality [10]. Globally, a 20-year analysis indicated a significant decrease in neonatal deaths from 400 to 210 per 100,000 live births between 1990 and 2010 [10].

Assessing the level of knowledge, attitudes, and practices of M and ON caring for pregnant patients can provide essential directions for nursing leadership to improve current care practices.

Specialized literature indicates that a lack of knowledge and limited access to the best evidence of care are the primary factors contributing to the ineffective management of pregnant patients in identifying and preventing pregnancy-related pathologies. A study in eastern South Africa identified deficiencies in midwives' knowledge of hypertensive conditions management during pregnancy [10].

The findings of Utz *et al*. [11] on the knowledge and practices of Moroccan general practitioners, midwives, and obstetric nurses caring for pregnant women with GD showed that they had a basic knowledge of the management of GD, while their reported practices were not uniform and reflected discrepancies with national guidelines in the field [11]. The study concluded that updating the knowledge of professionals in the field can result in more effective management of gestational diabetes (GD) within primary healthcare systems [11]. Another study conducted by Suff *et al*.[12] in the United Kingdom indicated a low level of theoretical and practical knowledge regarding the diagnosis and management of high blood pressure (Hbp) in pregnancy. Chepulis *et al*. conducted a study in New Zealand to assess the implementation of national screening guidelines for GD. The study found that the guidelines were not implemented in the sample of midwives included in the study, indicating a knowledge gap that may need to be addressed through education in this area [13].

The scoping review by Garti *et al*. using the three-step JBI methodology found similar results regarding a widespread lack of knowledge among practicing midwives globally in managing pregnancy-induced hypertension, as seen in the analysis of 29 studies. These findings highlight the need to develop accessible and innovative training programs for midwives and to establish policies that prioritize the professional development of midwives to increase their knowledge and skills in managing hypertension during pregnancy [14].

As per the current national legislation, midwives and nurses have a set of responsibilities which include identifying and planning care needs, patient education, monitoring vital functions, and treatment administration. Midwives and nurses are directly involved in the care of pregnant women with GD and PIH. They should possess comprehensive knowledge and practices to develop and apply an appropriate care plan for patients during antenatal and intrapartum periods. It is essential to screen pregnant women for GD and PIH from the first stage of hospitalization and to document the results of these evaluations in the patient's care plan. Providing health education to a pregnant woman with GD and PIH is necessary for the care given by M and ON and should include elements related to nutritional therapy, physical activity, and periodic monitoring of blood glucose and pressure. Maintaining blood glucose levels within normal limits is the main objective of care for pregnant women with GD. This can be achieved through interdisciplinary consultations with a diabetologist, nutritionist, or physiotherapist and by including the recommendations in the patient's care plan. The lack of a standardized protocol for providing maternal care to patients at risk can represent a significant barrier to the delivery of appropriate healthcare practices.

The main objective of our study was to evaluate the level of knowledge, attitudes, and practices of midwives and obstetric nurses at the Obstetrics and Gynecology Clinical Hospital Prof. Dr. Panait Sîrbu in providing care to pregnant women with GD and PIH. Our goal was to identify educational needs for developing a comprehensive training program to improve the care provided by healthcare providers. The second objective of our study was to assess the impact of a training program focused on GD and PIH on the level of knowledge, attitudes, and practices of midwives and obstetric nurses.

## MATERIAL AND METHODS

The study was designed as a longitudinal study with two primary objectives. The first objective was to develop an educational program that met the educational needs of M and ON, who provide direct care for pregnant patients with GD and Hbp. The second objective was to evaluate the impact of the educational program on the level of knowledge, attitudes, and practices of M and ON.

The study was conducted at the Obstetrics and Gynecology Prof. Dr. Panait Sîrbu Hospital, which provides medical services in obstetrics and gynecology, with an average of 531 hospitalizations per month, comprising both inpatient and outpatient care. The hospital has a capacity of 370 beds for inpatient care. In 2020, the total number of hospitalizations was 15,595, of which 4,836 were outpatient hospitalizations, 11,119 were inpatient hospitalizations, and 5,843 were deliveries. Of the total number of 234 midwives and obstetric nurses working in the hospital, 62 are midwives. To determine the appropriate population sample size for the study, calculations were made based on the total number of 234 professionals, with a confidence level of 95% and a 6% margin of error. As a result, 125 participants were randomly selected from the total number of M and ON who agreed to participate in this research.

Other categories of healthcare personnel, such as nurses working in the neonatology ward, physiotherapists, doctors, students, or other care staff, were excluded from the analysis.

To minimize the potential for bias, the research team maintained consistency in the study sample throughout all three stages of the study (M1, M2, M3), using the same group of 125 M and ON.

The study employed a quantitative research method to collect data on the knowledge, attitudes, and practices of M and ON working at a specialized hospital in Bucharest. The questionnaire was pre-tested and validated within a focus group of 25 experts in the field. We evaluated the questionnaire to assess its adaptability to the practices and competencies of M and ON in Romania, as well as the clarity of the text, readability, answer choice alternatives, contextual expressions, and the degree of difficulty of the items. The questionnaire used in our study consisted of a total of 56 questions that evaluated the practices, attitudes, and level of knowledge of midwives and obstetric nurses (M and ON) in managing gestational diabetes (GD) and pregnancy-induced hypertension (PIH). Specifically, the survey included 23 items about practices, 18 items about attitudes, and 15 items about knowledge of GD and PIH.

The research was conducted in 5 main stages. Firstly, the practices, attitudes, and knowledge of M and ON professionals caring for pregnant patients with GD and PIH (April 15, 2020) were assessed. Secondly, an educational program was developed based on the identified educational needs following the initial evaluation (moment 1 - M1) (May 1, 2020, to June 1, 2020). Thirdly, the educational program was delivered to a group of 125 M and ON professionals (July 1, 2020 - July 5, 2020). Fourthly, the practices, attitudes, and knowledge of M and ON professionals were evaluated immediately after completing the training program (moment 2 - M2) (July 5, 2020). Lastly, the practices, attitudes, and knowledge were reassessed 3 months after completion of the training program (moment 3 - M3) (October 1, 2020). The level of knowledge, attitudes, and practices of all M and ON who participated in the study were analyzed.

The educational program was drafted based on the educational needs identified among M and ON professionals included in the study at the beginning of the evaluation. The program included information on PIH, hypertension treatment, predictive tests for pathological increases in hypertension in the last trimester of gestation, the role of M and ON professionals in the follow-up and supervision of PIH, HELP syndrome, GD, and pregnancy, and its causes and effects on pregnancy, birth and delivery, the role of the M and ON in the care of patients with GD during pregnancy, during labor, and after delivery.

The data processing was performed using SPSS 20.0 program. To determine the statistical significance of the average scores in each of the three domains (practices, attitudes, and knowledge), we used analysis of variance (ANOVA). Additionally, we calculated Pearson correlation coefficients (r) to explore the relationship between the scores of the three domains and socio-professional characteristics such as age, education level, gender, professional experience, and place of work.

## RESULTS

The questionnaire was administered to 125 medical M and ON professionals working in the obstetrics and gynecology departments of the Clinical Hospital of Obstetrics and Gynecology Prof. Dr. Panait Sîrbu. The distribution of the sample by various characteristics is shown in Table 1. The average age of participants was 44.7 years, and the average professional experience was 18.2 years (Table 1).

**Table 1:** Socio-demographic characteristics.

Characteristics	N	%
**Age ^a^**		
20–29	8	6.4
30–39	19	15.2
40–49	55	44.0
50–59	38	30.4
≥60	2	1.6
No answer	3	2.4
**Gender**		
Female	124	99.2
Male	1	0.8
**Education level**		
Nurse with sanitary high school	5	4.0
Nurse with post-secondary health school	30	24.0
Nurse with a university education	26	20.8
Midwife	57	45.6
Nurse with master's degree	7	5.6
**Professional experience (years) ^b^**		
≤4	19	15.2
5–9	10	8.0
10–14	11	8.8
15–19	23	18.4
20–24	21	16.8
25–29	22	17.6
30–34	15	12.0
≥35	4	3.2
**Department**		
Obstetrics-gynecology	76	60.8
Delivery room	22	17.6
Intensive therapy	16	12.8
Outpatient	6	4.8
Emergency room	5	4.0

a – Average age=44.7; SD=8.2; ^b^ – Average professional experience=18.2; SD=10.3.

The evaluation of healthcare practices revealed a wide range of practices with relatively high variability during the initial phase of the research. The respondents reported rarely or never reading articles, journals, or books related to the research topics. Before participating in the educational program, the respondents recorded average scores for practices, attitudes, and knowledge. This suggests that despite 60.8% of respondents having attended training sessions on GD and PIH prevention and management, the level of knowledge, attitudes, and practices prior to the training was average, with correct answers varying between 52.8% to 92.8%.

The second phase of the study highlighted the significant impact of the training module on the participant's level of knowledge, attitudes, and practices. Specifically, there was a significant increase in the weights of responses indicating the permanent use of correct practices immediately after the training for all items assessed, and this increase was sustained three months after the training. However, one item related to the use of calibrated and well-maintained devices for measuring blood pressure showed a significant increase in the proportion of respondents who answered "always" immediately after the training. However, this difference was not significant when compared to the pre-training and 3-month post-training results.

The score for the practices scale was calculated by summing the correct answers for the 23 questions related to practices, resulting in a score that could theoretically range from 0 to 23. The scores were divided into 3 ranges: 0-8 points (low scores), 9-16 points (medium scores), and 17-23 points (high scores) for the graphic representation.

**Figure 1:**
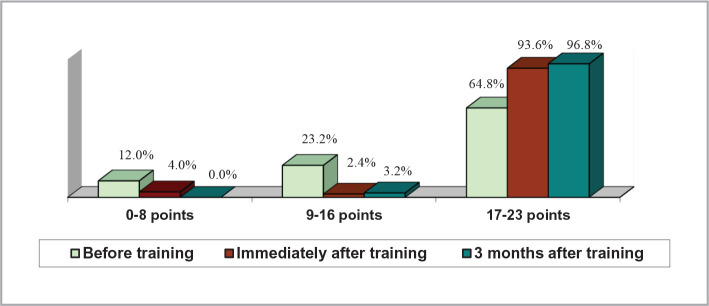
Practice scale scores.

The proportion of respondents who achieved high scores (between 17 and 23 points) on the practice scale increased from approximately 65% pre-training to over 93% immediately after and further increased to over 96% at 3 months follow-up (Figure 1). The average scores on the practice scale differed significantly between the three stages of the study, as indicated by Table 2.

**Table 2:** Comparison of average practice scores across three assessment stages.

	m	SD	Confidence Intervals 95%	df	ANOVA
**Before training (N=125)**	16.91	5.95	15.85–17.96	2	F=63.30**
**Immediately after training (N=125)**	21.79	3.34	21.19–22.38	2	
**3 months after training (N=125)**	22.13	1.98	21.78–22.48	2	

** – significant for p<0.01.

**Figure 2:**
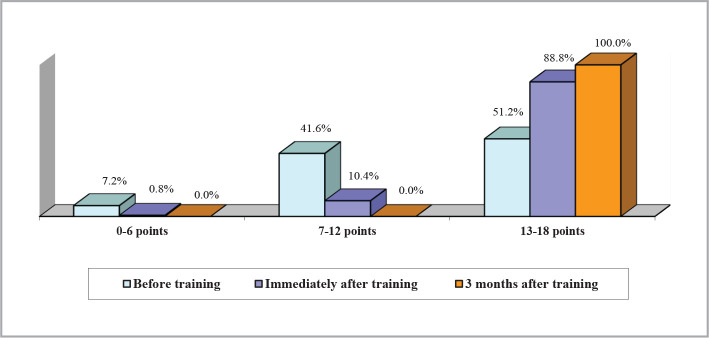
Attitude scale scores.

The attitudes of M and ON were evaluated in the three stages of the training program, and the percentage of high scores on the attitude scale significantly increased after the training, from approximately 51% to almost 89%. At both the immediate and 3-month follow-up assessments, all participants achieved scores within the high range of 13-18 points, as shown in Figure 2. The attitude score was calculated by summing the correct answers for the 18 attitude questions and could vary between 0 and 18. Scores were divided into three ranges: low (0-6 points), medium (7-12 points), and high (13-18 points) for the graphic representation.

The average scores also increased significantly, as confirmed by the analysis of variance (ANOVA) (Table 3).

**Table 3:** Comparison of average attitude scores across three assessment stages.

	M	SD	Confidence Intervals 95%	df	ANOVA
**Before training (N=125)**	12.08	3.50	11.46–12.69	2	F=148.26**
**Immediately after training (N=125)**	16.23	2.42	15.80–16.66	2	
**3 months after training (N=125)**	17.21	0.82	17.06–17.36	2	

** – significant for p<0.01.

**Figure 3:**
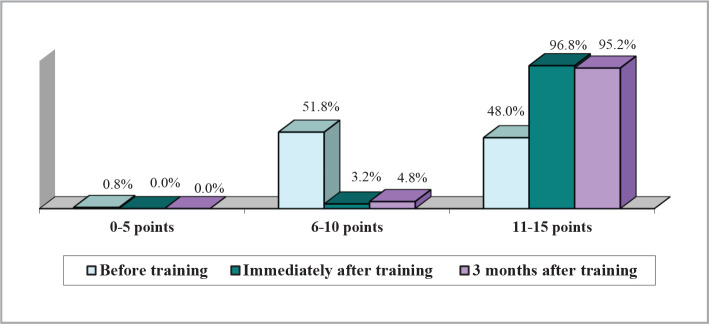
Knowledge scale scores.

The comparative assessment of the level of knowledge regarding PIH and GD at the three evaluation stages revealed some correct answers, but the proportion of correct responses significantly increased after completing the training module and remained significantly elevated three months after its completion compared to the pre-training stage, as illustrated in Figure 3.

Similarly, the score for the knowledge scale was determined by adding up the correct responses to the 15 questions, with possible scores ranging from 0 to 15. The scores were divided into 3 ranges: 0-5 points (low scores), 6-10 points (medium scores), and 11-15 points (high scores) for the graphic representation.

Figure 3 shows a significant increase in the percentage of high scores (11-15 points) on the knowledge scale, from 48%, before the training, to over 95% in the 2 subsequent stages of the training. This growth is also confirmed by the significant differences in the average scores between the pre-training and the other 2 post-training stages (Table 4).

**Table 4:** Comparison of average knowledge scores across three assessment stages.

	m	SD	Confidence Intervals 95%	df	ANOVA
**Before training (N=125)**	10.32	1.96	9.97–10.66	2	F=207.61**
**Immediately after training (N=125)**	14.12	1.26	13.89–14.34	2	
**3 months after training (N=125)**	13.50	1.42	13.25–13.75	2	

** – significant for p<0.01.

There is an exception recorded for item 18 regarding the definition of GD, for which the percentage of correct answers recorded is not significant. This result is likely because participants answered this item correctly in the pre-training stage. For the item related to the definition of proteinuria, there was no significant difference between the pre-training and 3-month post-training assessments, as the percentage of correct answers was consistently high (over 90%) across all three stages.

It is worth noting that for two other items (16 and 25, which concerned the value of blood pressure measurement for preeclampsia diagnosis and identifying non-risk factors for GD, respectively), the percentage of correct answers showed a significant decrease at the 3-month post-training assessment compared to the immediately post-training assessment. However, these percentages remained significantly higher than those at the pre-training stage.

Following the training program, all three scales were significantly and positively correlated. In contrast, prior to the training, no significant correlation was found between the attitude and practice scale scores, according to the data presented in Table 5.

**Table 5:** The correlation coefficients r between the scores of the 3 scales, age, and professional experience.

Item	Practice score	Attitudes score	Knowledge score	Age	Professional experience
**M1 – before training**					
Practice scale score	-	0.21	0.12*	0.26**	0.20**
Attitudes scale score		-	0.19*	0.04	0.01
Knowledge scale score			-	0.09	-0.004
Age				-	0.74**
Professional experience					-
**M2 – immediately after training**					
Practice scale score	-	0.41**	0.32**	0.12	0.24**
Attitudes scale score		-	0.38*	0.16	0.18*
Knowledge scale score			-	0.10	-0.14
Age				-	0.74**
Professional experience					-
**M3 – 3 months after training**					
Practice scale score	-	0.24*	0.48**	0.10	0.16*
Attitudes scale score		-	0.26**	0.14	0.18*
Knowledge scale score			-	0.04	-0.07
Age				-	0.74**
Professional experience					-

* – significant for p<0.05; ** – significant for p<0.01.

Participants reported the most frequently encountered challenges in providing appropriate care to patients with PIH or GD. In all 3 stages of the study, the participants identified the high volume of work and the lack of staff as the most common barriers.

However, the lack of healthcare protocols was also considered a significant obstacle to providing good care, particularly in the post-training stages. In contrast, the lack of courses dedicated to this topic was cited more frequently in the pre-training stage than in the post-training stages.

## DISCUSSION

The main objective of our study was to evaluate the knowledge, attitudes, and practices of midwives and obstetric nurses at the Obstetrics and Gynecology Clinical Hospital Prof. Dr. Panait Sîrbu in caring for pregnant women with GD and PIH. The evaluation was conducted at the 3 points: before the training, immediately after its completion, and three months later to assess the impact of the training on participants’ knowledge, attitudes, and practice. The comparative and correlational analysis of the three scales (practices, attitudes, knowledge) revealed a positive impact of the training program on M and ON practices, attitudes, and knowledge. This is further supported by the comparative analysis of the total scores of each set of items.

In light of the positive outcomes observed across all three evaluation scales (i.e., practices, attitudes, and knowledge) immediately after the training and at the three-month follow-up, the study also examined the correlation between age and professional experience. Before the training, only the practice scale scores had a significant positive correlation with age and professional experience. However, immediately after the training and at 3 months follow-up, both the practice and the attitude scale scores had a positive correlation with work experience, with higher scores reported among those with more experience. On the other hand, the knowledge scale did not show a significant correlation with age or professional experience. These findings highlight the positive impact of the training program on knowledge improvement, regardless of the participant's age or professional experience.

Comparative findings with our study have been reported by other researchers who assessed the level of knowledge in managing medical care for patients with PIH [10, 14-16] or GD [11, 13, 17]. A similar study conducted in a Romanian hospital initially revealed limited knowledge about preeclampsia and eclampsia among midwives and resident doctors caring for pregnant patients at different stages of pregnancy with PIH. Consequently, researchers recommended the development and promotion of studies that evaluate the impact of training sessions on this topic [18]. The development and implementation of an intensive training program focused on caring for patients with pregnancy-induced hypertension resulted in a significant improvement in the level of knowledge among the medical staff in the study. This finding is likely to benefit the care process provided by the medical staff [19]. Another study by Anyanti *et al*.[17] revealed a significant knowledge gap among health workers regarding the diagnosis and treatment of PIH and GD. The authors attributed this deficit to the lack of healthcare protocols tailored to the skills of medical staff in Nigeria [17]. The authors suggested the development of guidelines that highlight the specific role of each professional category involved in the care of patients with Hbp and GD and that specific work procedures should be available at the care points to ensure good treatment results [17].

Our results indicate that participants significantly improved their knowledge, attitudes, and practices regarding the care of patients with Hbp and GD after completing the training program. This improvement was sustained at a high level even after 3 months of completing the training program.

These results highlight the need for educational programs for M and ON, focused on the healthcare needs of pregnant patients at risk of developing GD and gestational Hbp. In addition to training programs, it is necessary to develop guidelines and work protocols for each category of personnel tailored to their specific skills and duties in the care process, as suggested by Anyanti *et al*. [17]. Our respondents indicated a lack of specific care protocols based on the best evidence of good practices in the field, which is a concern in Romania, given the differences in healthcare skills between midwives and obstetric nurses. In addition, implementing clinical audit activities on care protocols can represent an effective solution to ensure that the best care practices in this field are implemented in clinical practice. Another critical aspect of care during pregnancy is patient education. It is crucial to increase the parturient awareness of the possible risks that may develop during pregnancy. Patient education regarding GD and Hbp prevention plays an essential role in the care process provided by M and ON.

In a systematic review, Gholami *et al*.[20] investigated the effect of educational interventions on the knowledge of pregnant women regarding hypertensive disorders that may occur during pregnancy. The review included 6 studies on this topic, and the results of the analysis showed that multimodal patient education through educational brochures, mobile applications, combinations of brochures, iconographic images, videos, and PowerPoint presentations had a positive and significant effect on the awareness of pregnant women regarding the complications associated with hypertensive disorders of pregnancy. These findings suggest that providing adequate education for patients could reduce the number of severe complications caused by hypertensive disorders [20].

When implementing the best care practices for patients with gestational diabetes (GD) and hypertension (Hbp), healthcare professionals should consider both the facilitators and barriers that may impact the care provided by M and ON. In our study, participants identified high workload, doubled by the lack of staff and specific care protocols, as the main barriers in the care process.

A study in Ontario by Murray-Davis *et al*. assessed barriers and facilitators that may influence the improvement of midwife care practices for patients with GD or Hbp [21]. Midwives' behavior can be influenced by their knowledge, skills, social and professional role and identity, care context, and resources, especially when collaborating with other care providers. Integrating other specialists in the care of patients with GD or Hbp can improve midwives' experiences when providing specific care to patients with these pathologies [21].

PIH and GD are frequent complications associated with pregnancy at extreme ages and can have a negative effect from a medical, psychological, and social point of view, both for the parturient and the fetus. Periodic continuing medical education programs focused on this topic can improve the ability of midwives and obstetric nurses to identify these pathologies early and provide preventive healthcare. The positive effect of the training program on the practices, attitudes, and knowledge of M and ON identified in our study supports this conclusion. Additionally, implementing a healthcare protocol specifically designed for midwives and obstetric nurses on the care of pregnant patients at risk of developing these pathologies can be a viable solution for improving healthcare practices in obstetrics and gynecology departments across hospitals in Romania. Such protocols can also be valuable for professionals working in independent practice.

Furthermore, conducting clinical audits can be an effective way to ensure that healthcare protocols are being appropriately implemented in clinical practice. The study has some limitations due to the relatively small sample size and the fact that it was conducted only at a single specialized hospital. Subsequently, the conclusions of the study cannot be readily extended to all hospitals in Bucharest or the country.

## CONCLUSION

The results of our study highlight the importance of developing and implementing care protocols and educational programs for midwives and obstetric nurses to guide them in providing specific care for the prevention of Hbp and GD at all points of care. In addition, developing educational guidelines for pregnant patients can improve patient understanding of the risks associated with these conditions and increase compliance with care recommendations. Furthermore, our findings provide important evidence for healthcare leadership to support the value of continuing medical education in improving the medical practice of midwives and obstetric nurses.

## Personal thanks

We express our gratitude to Mrs. Ilona Voicu for her contribution to the statistical analysis of the data and Ms. Simona Rasu for providing English translation support, both of which were instrumental in the successful completion of this study.
